# *In vivo* antimalarial activity of the crude leaf extract and solvent fractions of *Croton macrostachyus* Hocsht. (Euphorbiaceae) against *Plasmodium berghei* in mice

**DOI:** 10.1186/1472-6882-14-79

**Published:** 2014-03-01

**Authors:** Laychiluh Bantie, Solomon Assefa, Tilahun Teklehaimanot, Ephrem Engidawork

**Affiliations:** 1 Department of Pharmacology and Clinical Pharmacy, School of Pharmacy, Addis Ababa University, P.O, Box 1176, Addis Ababa, Ethiopia; 2 Aklilu Lemma Institute of Pathobiology, Addis Ababa University, Addis Ababa, Ethiopia

**Keywords:** Antimalarial activity, *Plasmodium berghei*, *Croton macrostachyus*, *Chloroform fraction*, *In vivo* study

## Abstract

**Background:**

The issue of resistance in malarial infection makes development of novel drugs a necessity. An alternative source for discovering such drugs is natural products. *Croton macrostachyus* H. (Euphorbiaceae) is used in Ethiopian folklore medicine for the treatment of malaria and found to possess antimalarial activity *in vitro*. However, no further scientific investigations have been carried out to substantiate the claim. This study therefore aimed at investigating the *in vivo* antiplasmodial activity of 80% methanol extract and solvent fractions of the leaves of *Croton macrostachyus* H. in rodent model of malaria.

**Methods:**

A rodent malaria parasite*, Plasmodium berghei,* was used to inoculate healthy male Swiss Albino mice of age 6–8 weeks and weight 23–27 g. A hydro-alcoholic crude extract and the solvent fractions (chloroform, methanol and aqueous) were administered at different doses 200, 400 and 600 mg/kg. Parameters, including parasitemia, survival time, body weight, temperature, and packed cell volume were then determined using standard tests such as Peter’s and Rane’s test.

**Results:**

Chemoprotective effect exerted by the crude extract and fractions ranged between 44-91% and 12-76%, respectvely. The chemotherapeutic effect of the crude extract and chloroform fraction was in the range of 39-83% and 66-82%, respectively. Maximum effect in both tests was observed with the larger dose of the crude extract and chloroform fraction. The crude extract prevented loss of weight and reduction in temperature but did not affect packed cell volume. However, the chloroform fraction did also reverse reduction in packed cell volume due to the absence of saponins in the fraction.

**Conclusions:**

The results collectively indicate that the plant has a promising antiplasmodial activity against *Plasmodium berghei,* which upholds the earlier *in vitro* findings as well as its folkloric use. Thus, it could be considred as a potential source to develop new antimalarial agents.

## Background

Malaria is a major public health concern in the world, causing an estimated 0.7-1 million deaths per year. Approximately, half of the world’s population is at risk of malaria [1,2] and the vast majority of cases (78%) occur in the African region followed by Southeast Asia (15%) and Eastern Mediterranean regions (5%) [3]. Even though the disease is preventable and curable, it is still one of the greatest global public health challenges especially in sub-Saharan Africa [4].

There is broad consensus on the need for development of new agents, owing to the increasing resistance of the parasite to available agents. Antimalarial drug development can follow several strategies, ranging from minor modifications of existing agents to the design of novel agents that act against new targets [5]. Natural products are the source of the two most important drugs currently available to treat severe *falciparum* malaria, quinine and artemisinin derivatives. In case of artemisinin, relatively simple chemical modifications of the natural parent compound have led to the synthesis of a series of highly potent antimalarials [6,7]. The development of these two important drugs from natural sources and the utilization of many plants traditionally in various parts of the world triggered the search for new antimalarial drugs of natural origing using *in vitro* and *in vivo* studies.

*Croton macrostachyus* H. (Euphorbiaceae) is commonly found on forest edges along rivers, around lakes, woodlands, wooded grasslands and along roadsides. It is native to Eritrea, Ethiopia, Kenya, Nigeria, Tanzania and Uganda [8,9]. In Ethiopia, it is used for the treatment of malaria by the Shinasha, Agew-awi and Amhara people [10]. Recently, fourteen secondary metabolites have been isolated from *Croton macrostachyus* and five of them were isolated from the genus for the first time. The compunds include phenolics and triterpenes of the lupane and hopane groups [11].

Ethnobotanical/pharmacological studies revealed that *Croton macrostachys* has a wide range of activities. The plant has shown a promising antimicrobial [12], antidiabetic [8], purgative and anti-inflammatory effects [13]. The ethyl acetate extract of the stem bark [14] as well as the methanol and dichloromethane extracts of the leaves and stem bark of the plant have been demonstrated to possess significant antibacterial and antifungal activities [15,16]. An *in vitro* study of the fruit extract indicated that the plant had a good antiplasmodial activity [17]. Moreover, the methanol leaf extract of *Croton macrostachyus* exhibited larvicidal activity against late third instar larvae of *Anopheles arabiensis* Patton, a potent malaria vector in Ethiopia [18].

About 80% of the Ethiopian population use traditional medicine due to the cultural acceptability of healers, the relatively low cost of traditional medicine and the difficulty of accessing modern health facilities [19]. The studies conducted on the traditional medicinal plants in Ethiopia are, however, scanty when compared with the multiethnic cultural diversity as well as the diverse flora of Ethiopia. Although recently there are efforts to identify and screen antimalarial herbs used in the ethnomedicine practice of the country, the studies done are very limited and not fully exploratory. Most of them focused on reviewing the ethnobotanical uses of the plant rather than pharmacological screening [20]. Thus, based on ethnobotanical and *in vitro* studies mentioned above, the present study evaluated the *in vivo* antimalarial activity of the crude extract and solvent fractions of *Croton macrostachyus* H.

## Methods

### Plant material

Fresh leaves of Croton macrostachyus H. were collected in October 2010 from Shindi district of Amhara regional state, 420 km North West of Addis Ababa. The fresh leaves were wrapped with plastic sheets during transportation. The plant was identified as C*roton macrostachyus* by a taxonomist and a voucher specimen was deposited (No. LB 001) at the National Herbarium, College of Natural Sciences, Addis Ababa University for future reference.

### Animals and parasite

Male Swiss albino mice (age 6–8 weeks and weight of 23–27 g) bred and maintained at the Ethiopian Health Nutrition and Research Institute were used. They were maintained under standrad condition (temperature of 22 ± 3°C, relative humidity of 40-50% and 12 h light/12 h dark cycle), with food and water *ad libitum* in the animal house of Akililu Lemma Institute of Pathobiology, Addis Ababa University. Animals were acclimatized for one week to the experimental environment. The care and handling was according to international guidelines for the use and maintenance of experimental animals [21] and the School of Pharmacy Ethics committee approved the protocol.

Chloroquine sensitive strain of *Plasmodium berghei* (ANKA) obtained from the Department of Biology, College of Natural Sciences, Addis Ababa University was used. The parasites were maintained by serial passage of blood from infected mice to non-infected ones on weekly basis.

### Extraction

#### Crude extract

The leaves were air dried at room temperature under shade and reduced to appropriate size by grinding with an electric mill. A total of (600 g) dried leaves were extracted by maceration (100 g of dried leave in 600 ml of 80% methanol) for 72 h. The extraction process was facilitated by using an orbital shaker at 120 rpm. The mixture was first filtered using gauze and then with Whatman filters paper No. 1 (Whatman®, England). The residue was re-macerated for another 72 h twice and filtered. The combined filtrates were then dried by rotary evaporator (Buchi Rota vapor, Switzerland) at a temperature of 40°C. After drying, a total of 74.4 g of dry extract was harvested (18.6% yield) and the dried extract was kept at −20°C until use.

#### Solvent fractionation

The crude extract, after defatted with petroleum ether, was subjected to successive extraction in soxhlet apparatus using solvents with differing polarity (chloroform, absolute methanol and distilled water). The dried crude hydroalcoholic extract was placed in a cellulose thimble in an extraction chamber, which was placed on top of a collecting flask beneath a reflux condenser. Chloroform was first added to the flask, and the set up was heated under reflux. When a certain level of condensed solvent accumulated in the thimble, it was siphoned into the flask beneath and continued for 3 days [22] to get the chloroform fraction. The residue left in the thimble was next extracted using methanol following the same procedure as above to get the methanol fraction. The chloroform fraction was air dried at room temperature, while the methanol fraction was removed by using rotary evaporator. The residue left inside the thimble from the two solvent fractions was macerated in Erlenmeyer flask using distilled water to get the aqueous fraction. The aqueous fraction was then concentrated in a water bath and further dried using a lyophilizer (Operan, Korea vacuum limited, Korea). The yield of the dried fraction was 11.2%, 27.6% and 44.6% for the chloroform, methanol and aqueous fractions, respectively. The dried fractions were then transferred into separate vials and stored at −20°C until use.

### Acute toxicity testing

Twelve female Swiss albino mice were randomly divided into 2 groups of 6 mice per group. After being fasted for 2 h [21,23], mice in the first group were given 2 g/kg and the second group 5 g/kg of the crude extract orally and observed for any signs of toxicity daily for 14 days to assess safety of the extract. Animals were observed for gross changes such as loss of appetite, hair erection, lacrimation, tremors, convulsions, salivation, diarrhoea, mortality and other signs of overt toxicity [21].

### *In vivo* antimalarial tests

#### Parasite inoculation

Albino mice previously infected with *Plasmodium berghei* and having parasitemia level of 20-30% were used as donor. The donor mice were then sacrificed by decapitation and blood was collected by cardiac puncture into heparinized vacutainer tube containing 0.5% trisodium citrate. The blood was then diluted with physiological saline (0.9%) based on parasitemia level of the donor mice and the red blood cell (RBC) count of normal mice [24], in such a way that 1 ml blood contains 5 × 10^7^ infected RBCs. Each mouse was then given 0.2 ml of this diluted blood intraperitoneally, which contained 1 × 10^7^*Plasmodium berghei* infected RBCs.

#### Grouping and dosing of animals

For evaluating the crude extract, infected mice were randomly divided into five groups of 6 mice per group. Group I-III were treated with the crude extract of *Croton macrostachyus* at 200 mg/kg (CM200), 400 mg/kg (CM400) and 600 mg/kg (CM600), repectively. The remaining two groups served as negative and positive controls and administered distilled water (CON) (2 ml/100 g) [15] and chloroquine 25 mg/kg (CQ25), respectively.

The study on the fractions was conducted using thirty mice for each fraction. Mice were randomly assigned into three treatment groups and two controls, six mice per group for each fraction. Negative controls were administered the vehicle used for reconstitution (Tween 80 2% v/v in water for the chloroform and methanol fractions or distilled water for the aqueous fraction) (CON). Treatment groups were given the fractions at a dose of 200 mg/kg, 400 mg/kg and 600 mg/kg dissolved in the respective vehicle. The last group was treated with the standard, CQ25. Doses were selected based on acute toxicity studies. Volume administered was 0.2 ml and gavage was used for oral administration.

#### The 4 day suppressive test

This test was used to evaluate the schizontocidal activity of the extract and the fractions against *Plasmodium berghei* infected mice according to the method described by Peter et al. [25]. Infected mice were randomly divided into their respective group as described under grouping and dosing. Treatment was started three hours after mice had been inoculated with the parasite on day 0 and then continued daily for four days from day 0 to day 3. After treatment was completed, thin blood film was prepared from the tail of each animal on day 4 to determine parasitemia and percentage inhibition. In addition, each mouse was observed daily for determination of survival time.

#### Rane’s test

Evaluation of the curative potential of the crude extract and the most active fraction in Peter’s test was carried out according to the method described by Ryley and Peters [26]. On Day 0, standard inocula of 1 × 10^7^ infected erythrocytes were inoculated in mice intraperitoneally. Seventy-two hours later, mice were randomly divided into their respective groups and dosed accordingly once daily for five days. Geimsa stained thin blood film was prepared from the tail of each mouse daily for 5 days to monitor parasitemia level. Mean survival time for each group was determined arithmetically by calculating the average survival time (days) of mice starting from date of infection over a period of 30 days (D0-D29).

#### Packed cell volume measurement

Packed cell volume (PCV) was measured to predict the effectiveness of the test extract and fractions in preventing hemolysis resulting from increasing parasitemia associated with malaria. Heparinized capillary tubes were used for collection of blood from tail of each mouse. The capillary tubes were filled with blood up to ¾^th^ of their volume and sealed at the dry end with sealing clay. The tubes were then placed in a micro-hematocrit centrifuge (Gelma Awhksley, England) with the sealed end outwards and centrifuged for 5 min at 11,000 rpm. The tubes were then taken out of the centrifuge and PCV was determined using a standard Micro-Hematocrit Reader (Hawksley and Sons, England). PCV is a measure of the proportion of RBCs to plasma and measured before inoculating the parasite and after treatment using the following relationship:

$$\mathrm{PCV}=\frac{\mathrm{Volume}\;\mathrm{of}\;\mathrm{erythrocytes}\;\mathrm{in}\;a\;\mathrm{given}\;\mathrm{volume}\;\mathrm{of}\;\mathrm{blood}}{\mathrm{Total}\;\mathrm{blood}\;\mathrm{volume}}$$

#### Parasitemia measurement

Thin smears of blood were made from the tail of each mouse on day 4 for Peter’s test and on day 3–7 for Rane’s test. The smears were applied on microscope slides (76 × 26 mm) (Menzel-Glaser, Germany), fixed with absolute methanol for 15 min and stained with 10% Geimsa stain at pH 7.2 for 15 min. The stained slides were then washed gently using distilled water and air dried at room temperature. Two stained slides for each mouse were examined under Olympus microscope (CHK2-F-GS, Taiwan) with an oil immersion nosepiece of 100× magnification. Three different fields on each slide were examined to calculate the average parasitemia as shown below [27].

$$\%\;\mathrm{Parasitemia}=\frac{\mathrm{Number}\;\mathrm{of}\;\mathrm{parasitized}\;\mathrm{RBC}\;}{\mathrm{Total}\;\mathrm{number}\;\mathrm{of}\;\mathrm{RBC}}\times 100$$

Finally, percent parasitemia suppression of the extracts was compared with respect to the controls and parasitemia suppression was calculated using the following formula [28].

$$\%\;\mathrm{suppression}=\frac{\mathrm{mean}\;\mathrm{parasitemia}\;\mathrm{of}\;\mathrm{negative}\;\mathrm{control}-\mathrm{mean}\;\mathrm{parasitemia}\;\mathrm{of}\;\mathrm{treated}\;\mathrm{group}}{\mathrm{mean}\;\mathrm{parasitemia}\;\mathrm{of}\;\mathrm{negative}\;\mathrm{control}}\times 100$$

#### Monitoring of body weight and temperature changes

For Peter’s test, body weight of each mouse was measured before infection (day 0) and on day 4 using a sensitive digital weighing balance (Mettler Toledo, Switzerland). Rectal temperature was also measured by a digital thermometer before infection, four hours after infection and then daily. For Rane’s test, body weight and temperature were measured before infection and from day 3–7 after infection. In order to rule out the effect of the extract on body weight, temperature and PCV; the crude extract was administered to healthy mice at the doses used for four days. The extract was found to have no effect on temperature and PCV. However, the larger dose of the crude extarct (CM600) was found to significantly reduce body weight of healthy mice.

### Phytochemical screening

The crude extract and solvent fractions were screened for the presence of different secondary metabolites following standard procedures [29-32].

### Data analysis

Data are expressed as mean ± standard error of mean (SEM). Data were analyzed using Windows SPSS Version 16.0. One-way analysis of variance (ANOVA) followed by Tukey’s HSD *post-hoc* test was used to determine statistical significance for comparison of parasitemia suppression, weight, PCV, rectal temperature and survival time among groups. P-value of less than 0.05 was considered statistically significant.

## Results

### Acute toxicity study

The acute toxicity study indicated that the extract caused no mortality in both doses (2 and 5 g/kg) within the first 24 h as well as for the following 14 days. Physical and behavioural observations of the experimental mice also revealed no visible signs of overt toxicity like lacrimation, loss of appetite, tremors, hair erection, salivation, diarrhoea and the like. This suggests that LD50 of the extract is greater than 5 g/kg.

### Effect on four day suppressive test

#### Crude extract

The results of the study showed that the extract displayed chemosuppressive activity against *Plasmodium berghei* (Table 1). Percentage inhibition analysis indicated that the extract produced a dose-dependent decrease (p<0.001 in all cases) in level of parasitemia compared to CON mice. CM600 exhibited a significant parasite suppression compared to other doses of the extract and the activity was almost comparable to that of CQ25 (Table 1). The extract was also capable of significantly increasing survival time at all doses compared to controls, but the effect was significantly lower than CQ25.

**Table 1:** **Parasitemia and survival time of infected mice treated with crude extract and solvent fractions of *Croton macrostachys *** **in the 4 day suppressive test**

**Extract**	**Animal group**	**Parasitaemia level**	**% suppression**	**Survival date**
Crude extract	CON	32±0.97	-	7±0.36
	CM200	18±1.73	44^a3,b3,c3^	10±0.33^a1,b3,c3^
	CM400	7±0.31	78^a3,b1,c3^	14±0.42^a3,b3,c3^
	CM600	3±0.40	91^a3^	18±0.60^a3,c3^
	CQ25	0.0^a3^	100	30±0^a3^
Chloroform fraction	CON	30.43±0.61	-	7.34±0.42
	CF200	15.40±0.43	49.4^a3,c3^	9.00±0.68^c3^
	CF400	10.30±0.58	66.2^a3,c3,e3^	13.34±1.25^a3,c3,e1^
	CF600	7.34±0.15	75.9^a3,c3,d3,e3^	16.50±1.08^a3,c3,e3^
	CQ25	0.00±0.00	100.00^a3^	29.67±0.34^a3^
Methanol fraction	CON	34.33±1.62	-	6.84±0.17
	MF200	21.61±0.54	37.1^a3,c3^	8.83±1.14^c3^
	MF400	16.30±0.71	52.5^a3,c3,e2^	8.50±0.56^c3^
	MF600	12.28±0.52	64.2^a3,c3,d1,e3^	10.00±1.15^a1,c3^
	CQ25	0.00±0.00	100.00^a3^	30.00±0.00^a3^
Aqueous fraction	CON	37.47±0.84	-	7.00±0.25
	AF200	33.03±0.94	11.8^a2,c3^	7.84±0.60^c3^
	AF400	26.27±0.69	29.9^a3,c3,e3^	8.34±0.42^c3^
	AF600	22.94±0.79	38.8^a3,c3,d3,e3^	9.17±0.31^a2,c3^
	CQ25	0.00±0.00	100.00^a3^	30.00±0.00^a3^

Data are expressed as mean ± SEM; n = 6; a, compared to control; b, to 600 mg/kg; c, to CQ25mg/kg; d, to 400 mg/kg; e, 200 mg/kg: ^1^p<0.05, ^2^p<0.01, ^3^p<0.001; CF = Chloroform fraction, CQ = chloroquine, MF, methanol fraction, AF, aqueous fraction, CON = Control, CM, crude extract of *Croton macrostachys*. Numbers refer to dose in mg/kg.

Analysis of rectal temperature revealed that 80% methanolic extract of *Croton macrostachys* caused significant attenuation of reduction in temperature of *Plasmodium berghei* infected mice in a dose-dependent manner (p<0.01 for CM200 & CM400, p<0.001 for CM600). Both CM200 and CM400 had a comparable effect to CQ25, while CM600 appeared to have a better effect, although the result was not statistically significant (Table 2). The extract averted loss of weight associated with infection at all dose levels compared to CON mice. However, the increase in body weight was not found to be dose-dependent, as maximum effeect was obsreved with CM400 and minimum with CM200. There were no detectable differences in preventing weight reduction associated with parasitemia between the extracts as well as between the extract and CQ25 (Table 2). Neither the extract nor the standard prevented reduction of PCV when compared to CON animals.

**Table 2:** **Temperature, weight and packed cell volume of infected animals treated with crude extract and solvent fractions of *Croton macrostachys *** **in the 4 day suppressive test**

**Extract**	**Animal group**	**Temperature**			**Weight**			**Packed cell volume**		
		**D0**	**D4**	**% change**	**D0**	**D4**	**% change**	**D0**	**D4**	**% change**
Crude extract	CON	37.2±0.19	37.2±0.19	−2	26.0±0.18	24.0±0.43	−15.5	55±0.33	53±0.26	−3.6
	CM200	37.5±0.08	37.2±0.07	−0.8^a2^	26.1±0.12	25.3±0.34	−5.2^a1^	53±1.34	53±1.48	0.0
	CM400	37.1±0.11	37.1±0.11	0.5^a2^	26.2±0.11	25.9±0.15	−0.6^a3^	54±0.50	55±0.70	1.8
	CM600	37.0±0.15	37.0±0.15	1.3^a3^	26.2±0.17	25.7±0.22	−2.8^a2^	54±0.55	56±0.84	3.5
	CQ25	37.2±0.08	37.2±0.08	0.2^a2^	26.1±0.09	25.5±0.05	−2.4^a2^	53±0.55	55±0.42	3.8
Chloroform fraction	CON	37.3±0.42	32.9±0.21	−11.76	27.9±0.82	26.8±1.15	−4.00	53.3±1.46	49.7±1.61	−6.73
	CF200	36.9±0.37	36.3±0.47	−1.59^a3^	27.17±0.64	25.15±1.16	−7.43^b3^	51.7±1.78	51.1±1.1	−1.14^a1^
	CF400	37.5±0.32	37.4±0.23	−0.48^a3^	27.7±1.16	26.8±1.08	−3.82^b2^	52.2±1.25	52.2±1.16	0.00^a2^
	CF600	37.7±0.49	37.4±0.39	−0.93^a3^	28.1±1.20	27.9±1.12	−0.89^b1^	51.7±1.82	53.3±1.90	3.23^a3^
	CQ25	37.4±0.27	37.5±0.26	0.32^a3^	26.7±0.51	27.3±0.83	2.36^a2^	53.2±0.48	53.9±0.62	1.35^a2^
Methanol fraction	CON	37.3±0.21	35.4±0.17	−5.15	31.6±0.47	30.3±0.89	−3.83	53.8±1.61	52.3±1.05	−2.80
	MF200	37.7±0.29	35.2±0.71	−6.63^b3^	26.6±0.47	27.2±0.54	2.44^a3^	53.2±0.98	50.4±0.70	−5.17^b3^
	MF400	37.9±0.36	34.4±0.48	−9.24^b3^	26.8±0.55	28.1±0.59	4.43^a3^	52.8±0.96	51.2±0.90	−3.00^b2^
	MF600	38.0±0.15	34.7±0.23	−8.73^b3^	28.1±0.89	29.4±0.81	4.52^a3^	50.9±1.28	51.1±1.47	0.25
	CQ25	36.9±0.25	37.2±0.60	0.68^a3^	30.1±0.93	30.5±0.83	1.33^a2^	54.8±1.16	55.7±0.98	1.68^a1^
Aqueous fraction	CON	37.7±0.13	34.2±0.28	−9.30	31.7±0.57	30.4±0.65	−3.94	55.8±1.17	53.9±1.25	−3.40
	AF200	37.1±0.46	34.9±0.32	−5.90^b2^	30.8±0.79	30.7±0.54	−0.39^b1^	56.8±0.96	55.3±0.54	−2.64^b1^
	AF400	36.7±0.25	34.9±0.27	−5.20^b2^	29.2±0.86	28.9±0.75	−1.04^b1^	54.4±1.85	54.2±0.21	−0.46
	AF600	36.6±0.35	34.9±0.56	−4.97^b2^	30.0±1.04	29.0±0.90	−3.26^b2^	56.4±1.14	55.8±0.77	−1.19^b1^
	CQ25	36.9±0.35	36.6±0.26	−1.00^a3^	28.1±1.08	28.8±1.18	2.56^a2^	55.5±0.79	56.1±0.55	1.04^a1^

Data are expressed as mean ± SEM; n = 6; a = compared to control, b = to chloroquine 25 mg/kg; ^1^p< 0.05; ^2^p<0.01, ^3^p<0.001; D0 = pre-treatment value on day 0, D4 = post-treatment value on day four, CF = Chloroform fraction, CQ = chloroquine, MF, methanol fraction, AF, aqueous fraction, CON = Control, CM, crude extract of *Croton macrostachys*. Numbers refer to dose in mg/kg.

#### Solvent fractions

All the fractions dose-dependently reduced parasitemia level compared to CON group, although the extent of reduction was less than that of CQ25, which produced 100% suppression (Table 1). The rank order of chemosuppression of the solvent fractions was chloroform (75.9%) > methanol (64.2%) > aqueous (38.8%). Survival date was significantly prolonged (p<0.001) by the larger and middle doses of the chloroform fraction but only the larger dose of the methanol (p<0.05) and aqueous (p<0.01) fractions was capable of significantly increasing survival date compared to CON mice.

Body weight reduction caused by inoculation of the parasite was not significantly ablated by both chloroform and aqueous fractions. By contrast, all doses of the methanol fraction significantly prevented (p<0.001) body weight reduction. PCV reduction was dose-dependently attenuated by all doses of the chloroform fraction, but no detectable changes were noted with the other two fractions. Moreover, all doses of the chloroform fraction significantly (p<0.001) prevented the reduction in rectal temperature caused by escalating parasitemia and the effect was comparable to that observed with CQ25 mice (Table 2).

### Effect on curative test

#### Crude extract

The crude extract dose-dependently reduced (p<0.001 in all cases) parasitemia by 39, 69 and 83% for CM200, CM400 and CM600, respectively, compared to CON mice (Table 3). The inhibition obtained by CQ25 was significantly higher than CM200 (p<0.001) and CM400 (p<0.05) but, no apparent difference was observed with CM600. Survival time was not altered by CM200 but significantly increased by CM400 (p<0.001) and CM600 (p<0.001), however, the increase achieved with the extract was no match to that of CQ25 (Table 3). Rectal temperature analysis indicated that the extract had a significant preventive effect on the reduction in temperature at all doses compared to CON animals. Comparison of the doses amongst themselves as well as with CQ25, however, did not reveal any apparent changes (Table 4). Similar to the 4 day suppressive test, all doses of the extract failed to show any protective activity against the reduction in PCV (Table 4). The extract exhibited a preventive effect in weight reduction in infected mice at all dose levels compared to CON mice. However, the increase in body weight was not dose-dependent. No significant difference was noted in prevention of parasite-induced weight reduction when comparison was performed amongst the extract doses as well as with CQ25 (Table 4).

**Table 3:** **Parasitaemia and survival of infected animals treated with crude extract and chloroform fraction of *Croton macrostachys *** **in the Rane’s test**

**Extract**	**Group**	**Day 3**	**Day 4**	**Day 5**	**Day 6**	**Day 7**	**% inhibition**	**Survival date**
Crude extract	CON	9±0.15	16±0.13	14±0.23	31±0.61	36±1.1	-	6±0.31
	CM200	12±0.36	28±1.20	26±0.32	25±0.16	22±0.19	39^a3,b3,c3^	7±0.31^b3,c3^
	CM400	11±0.43	19±0.95	16±0.85	14±0.54	11±0.95	69^a3,c1^	12±0.47^a3,b3,c3^
	CM600	10±0.32	14±0.56	11±0.47	8±0.41	7±0.78	83^a3^	16±0.42^a3,c3^
	CQ25	11±0.22	5±0.09	2±0.56	0	0.00±0.00	100^a3^	30±0^a3^
Chloroform fraction	CON	21.8±0.58	26.6±0.90	33.1±0.81	40.7±0.67	54.6±1.34	-	7.2±0.31
	CF200	20.8±0.83	20.8±0.66	20.2±0.92	19.8±0.50	18.5±0.63	66.2^a3,c3^	7.5±0.34^c3^
	CF400	22.7±0.63	21.2±0.51	17.4±0.45	15.4±0.49	13.5±0.80	75.3^a3,c3,e2^	10.3±0.76^a2,d3^
	CF600	21.8±0.79	20.9±0.62	15.4±1.02	11.5±0.45	9.7±0.65	82.3^a3,c3,d1,e3^	12.3±0.72^a3,c3,e3^
	CQ25	20.8±0.85	15.6±1.02	5.5±0.28	1.5±0.29	0.00±0.0	100^a3^	30.0±0.0^a3^

Data are expressed as mean ± SEM; n = 6: a, compared to control; b, to 600 mg/kg; c, to CQ 25; d, to 400 mg/kg; e, to 200 mg/kg: ^1^p<0.05, ^2^p<0.01, ^3^p<0.001; CF = Chloroform fraction, CQ = chloroquine, MF, methanol fraction, AF, aqueous fraction, CON = Control, CM, crude extract of *Croton macrostachys*. Numbers refer to dose in mg/kg.

**Table 4:** **Rectal temperature, weight and packed cell volume of infected animals treated with crude extract of *Croton macrostachys *** **in the Rane’s test**

**Animal group**	**T0**	**T4**	**% change**	**W0**	**W4**	**% change**	**PCV0**	**PCV4**	**% change**
**CON**	37.4±0.14	36.6±0.05	−2.2	26.2±0.21	22.1±0.43	−7.7	54±0.31	53±0.21	−4.9
**CM200**	37.3±0.08	37.2±0.08	−0.2^**a2**^	26.3±0.11	25±0.16	−3.1^**a1**^	53±0.31	52±0.40	−1.9
**CM400**	37.4±0.09	37.4±0.04	0.0^**a3**^	26.4±0.11	26.3±0.05	−1.1^**a3**^	52.5±0.43	53±0.22	0.9
**CM600**	37.3±0.09	37.4±0.03	0.2^**a3**^	26.6±0.06	25.9±0.05	−1.8^**a2**^	52±0.31	53±0.44	1.9
**CQ25**	37.4±0.18	37.3±0.13	−0.2^**a2**^	26.5±0.04	25.9±0.04	−2.3^**a2**^	52.5±0.42	53±0.21	2.4

Data are expressed as mean ± SEM; n = 6; a,compared to controls, ^1^p<0.05, ^2^p< 0.01, ^3^p< 0.001; CM, crude extract of *Croton macrostachys;* W, weight; T, temperature; PCV, packed cell volume; 0 refers at day 0 and 4 to day 4. Numbers refer to dose in mg/kg.

#### Solvent fractions

The fraction with the highest antimalarial activity (chloroform fraction) in the four day suppressive test was further evaluated for its effect on established parasite infection. Chloroform fraction exhibited a dose-dependent reduction (p<0.001) of parasitemia compared to CON animals in the Rane’s test (Table 3). Maximum inhibition (82.3%) was attained with 600 mg/kg dose of the fraction. On the other hand, CQ25 cleared the parasite to undetectable level on day 7 and the reduction in parasitaemia was significantly higher (p<0.001) compared to CON as well as all doses of the fraction.

As shown in Table 3, lower dose of chloroform fraction was not associated with significant prolongation of survival time. Although the middle (p<0.01) and larger (p<0.001) doses of the fraction did significantly prolong survival time compared to CON mice, the effect was still much lower (p<0.001) than attained by CQ25. All doses of the fraction as well as CQ25 were shown to significantly (p<0.001) prevent the reduction in temperature compared to CON group. CQ25 halted temperature dropping significantly compared to CF200 (p<0.05). However, there was no significant difference compared to the middle and larger doses of the fraction (Table 5).

**Table 5:** **Effect of the chloroform fraction of *Croton macrostachys*** **on on rectal temperature of *Plasmodium berghei *** **infected mice in the Rane’s test**

**Group**	**Day 0**	**Day 3**	**Day 4**	**Day 5**	**Day 6**	**Day 7**
Control	37.25±0.21	34.05±0.15	34.08±0.19	33.48±0.26	33.53±0.21	32.75±0.16
CM200	37.48±0.3	33.38±0.21	34.48±0.33	34.76±0.27	35.62±0.26	36.43±0.24^a3,b1^
CM400	37.48±0.22	33.38±0.38	34.53±0.37	34.15±0.23	36.05±0.25	36.62±0.21^a3^
CM600	37.50± 0.21	33.64±0.32	33.91±0.26	35.74±0.14	36.50±0.28	36.91±0.13a3
CQ25	37.32±0.23	33.92±0.32	34.67±0.24	35.65±0.29	37.13±0.19	37.40±0.19^a3^

Data are expressed as mean ± SEM for six mice per group; a = compared to controls, b = to chloroquine 25 mg/kg; 1 = p<0.05; 3=p<0.001, CQ = chloroquine.

#### Phytochemical screening

Phytochemical screening of the hydroalcoholic crude extract of the leaves of *Croton macrostachyus* revealed the presence of alkaloids, saponins, phenolic compounds, cardiac glycosides, tannins, terpenoids and flavonoids. Anthraquinones and phlabotannins were, however, absent from the crude extract. Phytochemical composition of the different fractions is depicted in Table 6. The methanol fraction appeared to be rich in secondary metabolites compared to the other two fractions.

**Table 6:** **Phytochemical constituents of crude extract and solvent fractions of *Croton macrostachys***

**Secondary metabolites**	**Crude extract**	**Chloroform extract**	**Methanol extract**	**Aqueous extract**
Alkaloids	+	+	+	+
Saponins	+	-	+	+
Phenolic compounds	+	+	+	-
Cardiac glycosides	+	-	+	-
Tannins	+	-	+	+
Flavonoids	+	-	+	-
Terpenoids	+	+	-	-
Anthraquinones	-	-	-	-
Phlobatannins	-	-	-	-

-, absent; +, present.

## Discussion

The *in vivo* model was employed for this study because it takes into account the possible prodrug effect and possible involvement of the immune system in eradication of infection [24]. *Plasmodium berghei ANKA* was used in the prediction of treatment outcomes [33] and hence it was an appropriate parasite for the study. Moreover, several conventional antimalarial agents such as chloroquine, halofantrine, mefloquine and more recently artemisinin derivatives have been identified using rodent model of malaria [34].

The 4 day suppressive test, which mainly evaluates the antimalarial activity of candidates on early infections, and Rane’s test, which evaluates the curative capability of candidate extracts on established infections, are commonly used for antimalarial drug screening. In both methods, determination of percent inhibition of parasitemia is the most reliable parameter. A mean parasitemia level ≤ 90% to that of mock-treated control animals usually indicates that the test compound is active in standard screening studies [35].

It can be clearly seen from the results that percentage parasitemia measured in the 4 day test was reduced by the crude extract in *Plasmodium berghei* infected mice, pointing to the fact that the plant is endowed with antimalarial activity. Evidence comes for this assertion from studies that reported antimalarial activity of other species of the same genus such as *Croton zambesicus*[36]. Among the fractions, the chloroform and methanol fractions were found to possess higher blood schizontocidal activity than the aqueous fraction. This was evident from the chemosuppression obtained during the four day suppressive test, suggesting the possible localization of the active ingredients in these two fractions. Alkaloids, phenolic compounds and terpenoids present in these fractions could be responsible for their antimalarial activity. The alkaloids are known to possess antiplasmodial properties, the most famous being quinine. The highest antimalarial activity of the chloroform fraction was found to be similar with other reports that came out from *in vitro*[37] and *in vivo*[38] studies. The phytochemical constituents of chloroform and methanol fractions may have an individual or synergistic effect to exert their antimalarial activity. But traces may be found in the aqueous fraction, which could explain its lower antimalarial activity*.* Crude extract of *Croton macrostachys* is reported to possess *in vitro* antiplasmodial activity [17] as well as larvicidal property [18]. Moreover, antimicrobial activity has been attributed to the crude extract [15,16,39], where the chloroform and butanol fractions showing greater growth inhibitory, while the aqueous fraction lacking any growth inhibition effect [40]. Drugs with antibacterial activity such as tetracycline and its derivatives are employed in the treatment of malaria. These observations further reinforce the notion that compounds responsible for antimalarial activity of the plant might be concentrated in non-polar and semi-polar fractions.

Anemia, body weight loss and body temperature reduction are the general features of malaria-infected mice [41]. So, an ideal antimalarial agents obtained from plants are expected to prevent body weight loss in infected mice due to the rise in parasitemia. Despite the fact that the increase in weight was not consistent with increase in dose, the crude extract of *Croton macrostachys* significantly prevented weight loss associated with increase in parasitemia level. The preventive effect declined at the highest dose (CM600) which is consistent with the results of the parallel sub-acute toxicity study carried out to rule out the effect of the extract on the measured parameters. The fact that the preventive effect decreased with increasing dose might indicate that the plant could have appetite suppressive effect at higher doses. This appetite suppressive activity might be ascribed to saponins, flavonoids, glycosides and phenolic compounds found in the crude extract [42]. Unlike the crude extract, the chloroform and aqueous fractions did not protect animals from body weight loss but the methanol fraction did. This suggests the possibility of the localization of appetite suppressing components in these fractions [43] and nutrients and other immunomodulatory substances in the methanol fraction [33].

A decrease in the metabolic rate of infected mice occurs before death and is accompanied by a corresponding decrease in internal body temperature [44]. Ideally, the rectal temperature decreases as parasite level escalates. Active compounds should prevent the rapid dropping of rectal temperature. The crude extract and the chloroform fraction did have protective effects against temperature reduction, which reflects constituents responsible for this effect were likely found in this fraction.

PCV was measured to evaluate the effectiveness of the crude extract and fractions in preventing hemolysis due to escalating parasitaemia level. The underlying cause of anemia includes the following mechanisms; the clearance and/or destruction of infected RBCs, the clearance of uninfected RBCs, and erythropoietic suppression and dyserythropoiesis. Each of these mechanisms has been implicated in both human and mouse malarial anemia [45]. One could note that the chloroform fraction was the only one that ablated PCV reduction. This observation is consistent with studies conducted on chloroform fraction of other plants, including *Dodonea Angustifolia*[46]. Failure of the crude extract and the two fractions to reverse PCV reduction could probably be related to the presence of saponins in the extract, which are known to have strong hemolytic effects [46].

In the curative test, blood samples were taken and smears prepared daily to evaluate the curative ability of the extract. As indicated in the results section, all doses of the crude extract and fractions brought about reduction of parasitemia after second dose, however, the standard drug chloroquine started its activity right after the first dose. This delay of activity may be indicative of the need for a loading dose or the extract might have a delayed onset of action. The curative effect of chloroform fraction occurred in a dose-dependent manner, with the highest suppression observed with its higher dose, and this is in line with previous reports [38,47]. Furthermore, the chloroform fraction prolonged the mean survival time in established parasite infection, which is also concordant with the study conducted on *Melanthra scandens*[38]. Taken together, the results obtained from the Rane’s test suggest that the chloroform fraction has therapeutic efficacy against established malaria parasite. This property is additive to the suppressive activity and it may be possible to consider the plant as a potential source of antimalrial agents, as it is desirable to have both activities in a potential phytodrug [48].

Although the active compound is yet to be identified, the antimalarial activity of *Croton macrostachyus* could be attributed to a single or a combination of its secondary metabolites such as alkaloids, flavonoids, terpenoids and phenolic compounds. These metabolites have been reported to have different extent of antimalarial activity in the literature [49-51]. Many species of the genus *Croton* were also reported to have promising antimalarial activity in different *in vitro* and *in vivo* studies [52,53].

*In vivo* antiplasmodial activity can be classified as moderate, good, and very good if an extract displayed percentage parasitemia suppression equal to or greater than 50% at a dose of 500, 250 and 100 mg/kg body weight per day, respectively [54,55]. Based on this classification, the crude extract as well as the chloroform and methanol fractions of the studied plant showed good antiplasmodial activity.

## Conclusions

The present study indicates that 80% methanolic extract and solvent fractions of *Croton macrostachys* have good antiplasmodial activity, with varying degree and/or differential effect on the measured parameters. The crude extract appeared to be superior in supressing parsitemia but was devoid of effect in protecting infected animals from parasite-induced PCV reduction. Although the chloroform fraction protected PCV reduction and displayed greater parasite suppression among the fractions, it failed to have a benefcial effect on body weight reduction. The findings suggest that the phytochemicals responsible for antimalarial activity of the plant are non-polar to semi-polar in nature and future studies on the plant regrading antimalarial activity should be conducted using the crude extract. In addition, the data would provide evidence to uphold the earlier *in vitro* findings as well as the claims made by the Ethiopian traditional medicine practioners.

## Competing interests

We declare that there are no conflicts of interest to disclose.

## Authors' contributions

LB and SA conducted the actual study and the statistical analysis. TT and EE were involved in developing the idea and desgining of the study. LB and EE were also involved in the write up of the manuscript. All authors approved the submitted version of the manuscript.

## Pre-publication history

The pre-publication history for this paper can be accessed here:

http://www.biomedcentral.com/1472-6882/14/79/prepub
